# Whole-genome sequencing for antimicrobial surveillance: species-specific quality thresholds and data evaluation from the network of the European Union Reference Laboratory for Antimicrobial Resistance genomic proficiency tests of 2021 and 2022

**DOI:** 10.1128/msystems.00160-24

**Published:** 2024-08-06

**Authors:** Lauge Holm Sørensen, Susanne Karlsmose Pedersen, Jacob Dyring Jensen, Niamh Lacy-Roberts, Athina Andrea, Michael S. M. Brouwer, Kees T. Veldman, Yan Lou, Maria Hoffmann, Rene S. Hendriksen

**Affiliations:** 1 National Food Institute, Technical University of Denmark, Research Group for Global Capacity Building, European Union Reference Laboratory for Antimicrobial Resistance, Kongens Lyngby, Denmark; 2 Wageningen Bioveterinary Research part of Wageningen University and Research, Lelystad, the Netherlands; 3 U.S. Food and Drug Administration, Center for Food and Safety and Applied Nutrition, College Park, Maryland, USA; London School of Hygiene & Tropical Medicine, London, United Kingdom

**Keywords:** whole-genome sequencing, quality control, short-read sequencing, antimicrobial resistance, genomic proficiency test, statistical comparison, next-generation sequencing

## Abstract

As antimicrobial resistance (AMR) surveillance shifts to genomics, ensuring the quality of whole-genome sequencing (WGS) data produced across laboratories is critical. Participation in genomic proficiency tests (GPTs) not only increases individual laboratories' WGS capacity but also provides a unique opportunity to improve species-specific thresholds for WGS quality control (QC) by repeated resequencing of distinct isolates. Here, we present the results of the EU Reference Laboratory for Antimicrobial Resistance (EURL-AR) network GPTs of 2021 and 2022, which included 25 EU national reference laboratories (NLRs). A total of 392 genomes from 12 AMR-bacteria were evaluated based on WGS QC metrics. Two percent (*n* = 9) of the data were excluded, due to contamination, and 11% (*n* = 41) of the remaining genomes were identified as outliers in at least one QC metric and excluded from computation of the adjusted QC thresholds (AQT). Two QC metric correlation groups were identified through linear regression. Eight percent (*n* = 28) of the submitted genomes, from 11 laboratories, failed one or more of the AQTs. However, only three laboratories (12%) were identified as underperformers, failing across AQTs for uncorrelated QC metrics in at least two genomes. Finally, new species-specific thresholds for “N50” and “number of contigs > 200 bp” are presented for guidance in routine laboratory QC. The continued participation of NRLs in GPTs will reveal WGS workflow flaws and improve AMR surveillance data. GPT data will continue to contribute to the development of reliable species-specific thresholds for routine WGS QC, standardizing sequencing data QC and ensure inter- and intranational laboratory comparability.

**IMPORTANCE:**

Illumina next-generation sequencing is an integral part of antimicrobial resistance (AMR) surveillance and the most widely used whole-genome sequencing (WGS) platform. The high-throughput, relative low-cost, high discriminatory power, and rapid turnaround time of WGS compared to classical biochemical methods means the technology will likely remain a fundamental tool in AMR surveillance and public health. In this study, we present the current level of WGS capacity among national reference laboratories in the EU Reference Laboratory for AMR network, summarizing applied methodology and statistically evaluating the quality of the obtained sequence data. These findings provide the basis for setting new and revised thresholds for quality metrics used in routine WGS, which have previously been arbitrarily defined. In addition, underperforming participants are identified and encouraged to evaluate their workflows to produce reliable results.

## INTRODUCTION

Compared to classical biochemical methods, the declining price and relatively expeditious workflow of whole-genome sequencing (WGS) constitutes an appealing alternative approach to conducting bacterial and viral typing (1–3). The amount of data produced and, by extension, the possibilities for rapid further analysis makes it a powerful tool. In antimicrobial resistance (AMR) surveillance, WGS allows high resolution of circulating genes and phylogenetic characterization, useful in outbreak detection and retrospective analysis. This enables better understanding and tracking of AMR transmission events and dissemination. For these reasons, WGS have been applied in a wide range of areas, including clinical settings, primary food production, and household environments (4–7).

Second- or next-generation sequencing (NGS) platforms marked a paradigm shift in sequencing through mass parallelization. NGS platforms increased throughput exponentially at a significantly lower cost than the prior sequencing technologies (8, 9). The continual trend of reduced NGS prices (https://www.genome.gov/about-genomics/fact-sheets/DNA-Sequencing-Costs-Data) has facilitated the implementation of WGS strategies in AMR surveillance (10), which is now feasible even in low- and middle-income country settings (11, 12).

Presently, we are witnessing the ongoing development of long-read third-generation sequencing technologies (13). Third-generation sequencers can generate ultra-long reads with an average length of 20 kb in real time (13, 14). High error rates in long-read sequencing technology pose a challenge for accurate genomic analysis. Thus, NGS is still the most widely used technology for bacterial sequencing, with WGS and AMR surveillance sequence data being mainly produced on Illumina platforms (15–17).

The continuing development and wide range of applications in sequencing have resulted in the emergence of numerous laboratory workflows for WGS, along with bioinformatics pipelines to conduct the subsequent data handling and analysis. These diverging developments pose challenges to standardization of quality control (QC), curation, and analysis (18, 19). While currently established QC metrics, such as N50 and number of contigs, are widely used, thresholds for good quality are poorly or arbitrarily defined. This is in part due to large inter- and intraspecies variation, though efforts have been made to define more precise QC boundaries at the species level (20–22).

To accommodate this issue, ongoing inter-laboratory proficiency tests can help to identify pitfalls in a laboratory WGS workflow and evaluate a laboratory’s capabilities in producing consistently high-quality WGS data, essential for conducting reliable data analysis. The EU Reference Laboratory for Antimicrobial Resistance (EURL-AR) genomic proficiency test (GPT), conducted by the Research Group for Global Capacity Building at the National Food Institute, Technical University of Denmark (DTU), aims to assess the ability of participating laboratories to conduct reliable WGS and AMR data analysis. Building upon findings in the first iteration of the GPT conducted in 2020 (21), we hereby assess the ability of the national reference laboratories (NRLs) that participated in the 2021 and 2022 iterations of the GPT. We look at their ability to conduct WGS based on a statistical approach to estimate QC thresholds. Furthermore, we evaluate relevant QC parameters used for comparing sequencing QC using linear regression to investigate possible correlation. Finally, we aim to improve and update the quality thresholds for the bacterial species included in the GPT based on the QC thresholds obtained in the 2020 iteration.

## MATERIALS AND METHODS

### Test material and collected data

Each year, six isolates of three relevant bacterial species were sent to participants as live culture (BACT) and as purified DNA (pDNA) resulting in a total of 12 samples per year for each participating NRL. Participants could freely select the species and sample types that were relevant for their laboratory during registration. Samples were prepared as described in the 2020 iteration (21), with the amendment of DNAstable not being added to pDNA samples. An overview of the isolates included in the EURL-AR GPT 2021 and 2022 is presented in Table 1.

**TABLE 1:** Reference strains used in the GPT 2021 and 2022^*a*^

ID	Species	Sequence type	Total base pairs	No. of plasmids	Source (sample accession)
GPT21-001	*Salmonella enterica* serovar Thyphimurium	34	5,367,509	4	This paper (GCA_963575755)
GPT21-002	*Salmonella enterica* serovar Infantis	32	4,967,824	1	This paper (GCA_963575775)
GPT21-003	*Escherichia coli*	641	4,923,186	4	This paper (GCA_963575765)
GPT21-004	*Escherichia coli*	21	5,560,407	5	This paper (GCA_963575795)
GPT21-005	*Campylobacter coli*	1017	2,018,501	2	This paper (GCA_963575805)
GPT21-006	*Campylobacter coli*	860	1,740,899	0	This paper (GCA_963575785)
GPT22-001	*Staphylococcus aureus*	398	2,934,440	3	This paper (GCA_029094305)
GPT22-002	*Staphylococcus aureus*	398	2,915,959	2	This paper (GCA_029094145)
GPT22-003	*Escherichia coli*	405	5,490,800	3	This paper (GCA_029094425)
GPT22-004	*Escherichia coli*	410	5,164,418	6	This paper (GCA_029094485)
GPT22-005	*Enterococcus faecium*	1424	3,234,880	8	This paper (GCA_029167665)
GPT22-006	*Enterococcus faecalis*	6	3,407,461	3	This paper (GCA_029167565)

^*a*^ Reference strains used in the DTU GPT 2021 and 2022, MLST, total genomic number of base pairs, and number of plasmids.

The laboratories conducted WGS and analysis of isolates using their routine workflows. Raw sequence data (fastq files) and details on applied methodologies were returned to EURL-AR for QC analysis. AMR detection and predicted AMR profiles were reported separately and will not be presented in this study, but published at a future date.

### Reference material

For each yearly iteration, a closed reference genome was produced for each isolate. In 2021, the reference genomes were produced at the Wageningen Bioveterinary Research (WBVR), from hybrid assemblies of Illumina Nextseq paired-end short reads, provided by the EURL-AR and long-reads, produced at WBVR. Long reads were generated using the MinIon Mk1C (Oxford Nanopore Technologies, UK) using the ligation sequencing kit SQK-LSK109 with Native Barcoding and flowcell R9.4.1. Long reads were basecalled using the high accuracy model in Guppy v4.4.1 and filtered to a minimal length of 500 bp using Filtlong v0.2.0. Short- and long reads were combined via hybrid assembly using Unicycler v0.4.8 (23) with dependencies SPAdes v 3.13.1 (24), Miniasm v 0.3-r179 (25), and Racon v 1.3.3 (26). Contigs that were not yet fully closed were curated manually using assemblies based only on long-read data.

In 2022, closed references were produced at the Center for Food Safety and Applied Nutrition, U.S. Food and Drug Administration (FDA). Multiplexed microbial SMRTbell libraries were made with the SMRTbell prep kit 3.0 following the PacBio protocol (PacBio, Menlo Park, CA, November 2021). The multiplexed SMRTbell library was then sequenced on Sequel IIe system (PacBio, Menlo Park, CA) using Binding Kit 3.2 and Sequel sequencing kit 2.0 on one SMRT cell 8M (PacBio, Menlo Park, CA), with 30 hours collection time. Raw HiFi reads were demultiplexed by running the Demultiplex Barcodes application and *de novo* assembled using the microbial genome analysis (MGA) in SMRTLink (PacBio, Menlo Park, CA). The final assemblies generated by MGA were then circularized and oriented to start at oriC.

### WGS reads processing

**Fig 1:**
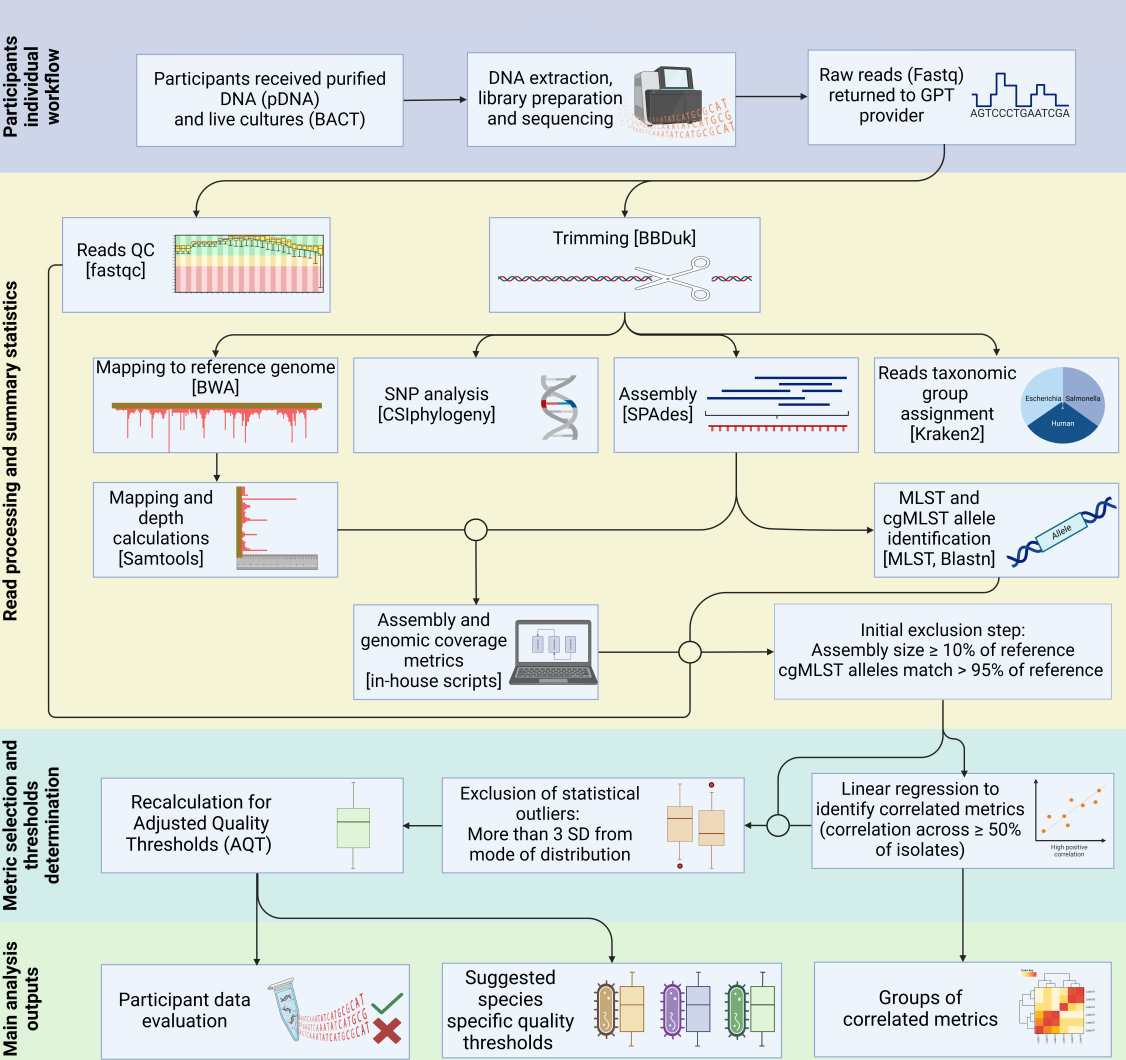
Analysis workflow. Overview of the sequence data generation and analysis. Square brackets indicate the tool used for the analysis step. Circles indicate inputs from multiple previous steps. SD, standard deviations. Created with BioRender.com.

Raw Illumina sequence data submitted by participants was processed as described in the GPT 2020 iteration (21) with the amendment that assemblies were constructed with SPAdes v 3.15.3 (24). An overview of data processing and analysis is presented in Fig. 1. Summary QC statistics were calculated with revised in-house Python3 pipelines.

### Typing and SNP distance

Single nucleotide polymorphism (SNP) distances were checked with CSIphylogeny (27), with genomes from the same isolate expected to have 10 or less SNPs between reference and corresponding participant genomes, changed from five SNPs in the 2020 iteration. Sequence type was determined for genomes and closed references using schemes for multilocus sequence typing (MLST) *in silico* using MLST v 2.0.4 (28). An additional change from the 2020 iteration was the inclusion of core genome MLST (cgMLST) genes. Alleles were downloaded from the cgMLST nomenclature server (https://www.cgmlst.org), identified with Blastn v 2.13.0+ (29) and compared with those in the reference genomes.

### Linear regression

**Fig 2:**
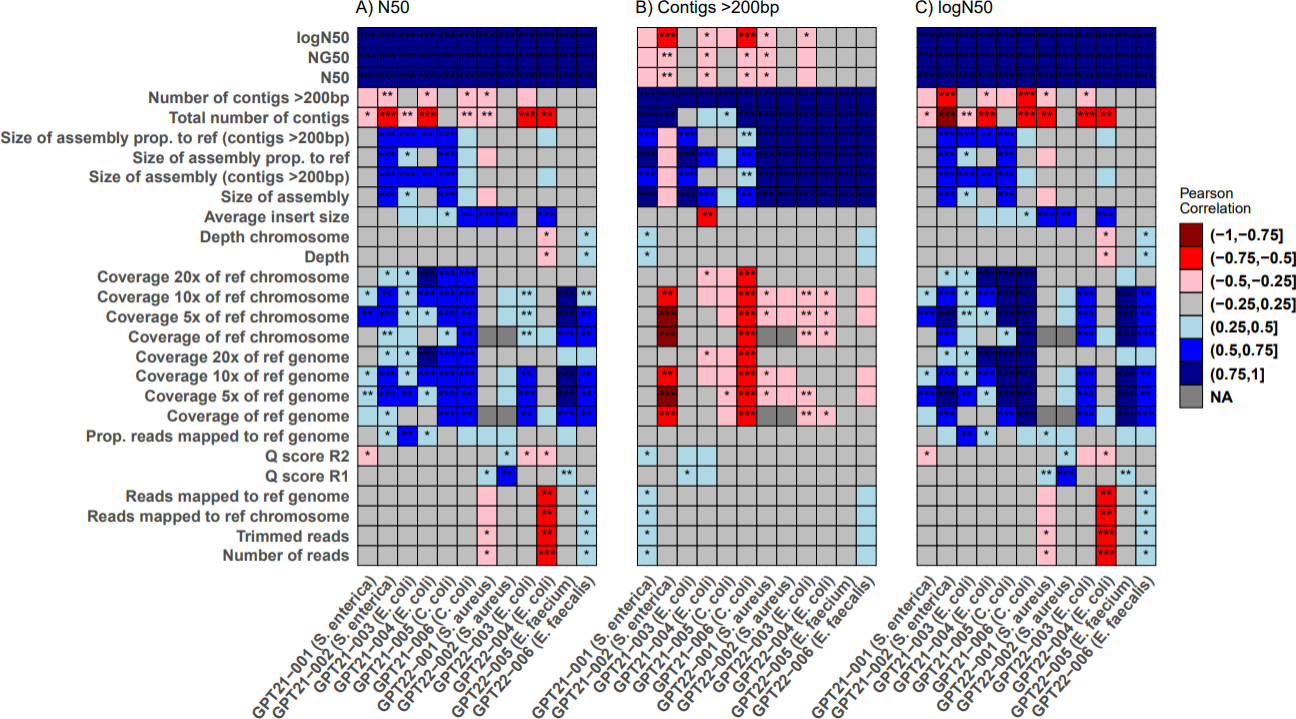
Metric correlation heatmaps. Heatmaps showing Pearson correlation between “N50” (left), “contigs > 200 bp” (middle), and “logN50” (right). *P*-value is indicated with stars: *<0.05, **<0.01, ***<0.001. Color indicates level of correlation, with red and blue signifying negative and positive correlation, respectively. Only laboratories outside AQT have been labeled. Data points removed in the initial exclusion step are not shown.

A linear regression analysis was conducted in R as previously described (21) using 27 metrics (see Fig. 2), with the following deviation. BACT and pDNA genomes were pooled before regression. Distributions for BACT and pDNA were compared with Welch *t*-test or Kolmogorov–Smirnov test (see Table S1). Sequences were then subjected to an initial exclusion step, sequences that produced assemblies that deviated more than 10% in size compared to the corresponding reference or which did not match 95% of its cgMLST alleles were eliminated. Metrics showing significant correlation in half or more of the distributed strains were considered further.

### QC analysis

Statistical analysis was performed on the data passing the initial exclusion step with R and plotted using the libraries ggplot2, stringr, broom, dplyr, reshape2, and data.table. QC parameters were evaluated in two steps: first an initial outlier identification, followed by a low-performance identification step based on recalculated thresholds, excluding identified outliers. This second step is referred to as adjusted quality threshold (AQT) below. Thresholds for outlier detection and AQTs were set using a statistical approach of three standard deviations from the estimated mode of the distribution (21). The estimation of the mode was changed from mean to median for “N50” and “Number of contigs > 200 bp.” Thresholds for “N50” were based on the natural logarithmic transformed data set to reduce variance. See Table 2 for parameters and methods for calculating thresholds.

**TABLE 2:** Parameters and threshold calculation methods^*a*^

Parameter	Outlier and adjusted quality threshold calculation
Percentage coverage of minimum 10× depth of the reference genome	More than 3 standard deviations below the mean
Proportion of reads mapped to the reference genome	More than 3 standard deviations below the mean
N50	More than 3 standard deviations below the median of the log-transformed data set
Size compared to reference genome	More than 3 standard deviations from the mean
Number of contigs >200 bp	More than 3 standard deviations above the median
Phred quality score (forward and reverse read separately)	More than 3 standard deviations below the mean

^*a*^ QC metrics evaluated in the DTU genomic proficiency test and the respective method for calculating the quality thresholds. The same method was applied after outlier removal to set AQT.

If participants had two or more genomes that were either below AQT across QC correlation groups or were removed in the initial exclusion step, their WGS results were deemed generally underperforming, and they were strongly recommended to review their laboratory workflows. The data evaluation workflow is summarized in Fig. 1. Kraken2 (30) was run on trimmed fastq files to estimate any possible contamination. Participating laboratories were numbered according to their numbering in the DTU GPT 2020 publication, to allow ease of comparison between years (21). Suggested species-specific QC thresholds were calculated by taking the average of obtained AQTs for each individual species.

## RESULTS

### Participation and reported methodology

In 2021 and 2022, 16 and 19 EURL-AR laboratories participated, respectively, consisting of 25 unique participating NRLs. These represent the EU NRLs of Austria, Belgium, Cyprus, Czech Republic, Denmark, Estonia, Finland, France, Germany, Ireland, Italy, Latvia, Lithuania, Luxembourg, Netherlands, Portugal, Slovakia, Slovenia, Spain, and Sweden and NRLs of the European Free Trade Association countries Norway and Switzerland. In total, 22 countries were represented in the 2021 and 2022 iterations of the GPT (see Fig. S1).

Most laboratories utilized manual DNA extraction in both years (2021: *n* = 10, 62.5%; 2022: *n* = 12, 63.2%). In 2021, two laboratories used a different extraction technique for *Campylobacter* spp. one opting for manual extraction, the other for automatic.

Fluorometers (Qubit [Thermo Fisher], Quantus [Promega], or Quant-it [Thermo Fisher]) were used by nearly all laboratories to quantify DNA in samples, whereas a few laboratories in 2021 (*n* = 2, 12.5%) and 2022 (*n* = 3, 15.8%) used spectrophotometers QIAxpert (Qiagen) or Nanodrop (Thermo Fisher) instead. Gel electrophoresis was only used in 2021 to quantify pDNA (*n* = 1, 6.3%). In both 2021 and 2022, one laboratory did not quantify DNA for *E. coli*. One laboratory did not quantify live culture *Campylobacter* in 2021, and one did not in 2022 for pDNA of *Enterococcus*.

DNA library preparation kits used in 2021 were predominantly Illumina DNA Prep (*n* = 8, 50%), followed by Illumina Nextera XT library kit (*n* = 3, 18.8%) and Nextera DNA Prep (*n* = 2, 12.5%). The three remaining laboratories used Illumina TruSeq PCR Free, NEBNext Ultra II FS DNA Prep, or did not report the kit (*n* = 1, 6.3%). In 2022, Illumina DNA Prep continued to be mainly used (*n* = 10, 52.6%), followed by Illumina Nextera XT (*n* = 3, 15.8%). Other kits included Celero EZ DNA-Seq, Collibri ES DNA Prep, NEBNext Ultra DNA Prep, NEBNext Ultra II FS DNA Prep, and Illumina Nextera DNA Prep (each kit *n* = 1, 5.3%). One participant reported using Illumina DNA Prep for GPT22-005 (*E. faecium*), but Celero EZ DNA-Seq for all other samples.

Among utilized platforms, MiSeq was the most commonly used in both years (2021: *n* = 10, 62.5%, 2022: *n* = 10, 52.6%). NextSeq was used more in 2022 (2021: *n* = 2, 12.5%, 2022: *n* = 4, 15.8%), and ISeq was used by two participants, one used it for all isolates, and the other only for *Enterococcus* isolates (2021: *n* = 2, 12.5%, 2022: *n* = 2, 10.5%). The NovaSeq6000 was used by one more laboratory in 2022 (2021: *n* = 2, 12.5%, 2022: *n* = 3, 15.8%), and the MiniSeq was used by a single laboratory in 2022 (*n* = 1, 5.3%).

### Evaluation of sequence data

In the initial exclusion, two genomes from 2021 and seven from 2022 were removed from the data set of 180 genomes in 2021 and 212 genomes in 2022. Of these nine genomes, six were removed due to exceeding the expected assembly size by more than 10% and three due to <95% match to the expected cgMLST alleles. The excluded genomes were submitted by the NRLs Lab01, Lab07, Lab12, Lab13, Lab16, Lab18, Lab19, and Lab25. Each of these participants had one genome excluded, with the exception of Lab13, which had two genomes excluded, listed in Table S2. The remaining numbers of strains after exclusions are presented in Table 3.

**TABLE 3:** Genome counts after initial exclusion^*a*^

	Species	Strain code	pDNA	BACT	Total
GPT 2021	*S. enterica*	GPT21-01	15	16	31
	*S. enterica*	GPT21-02	15	16	31
	*E. coli*	GPT21-03	14	15	29
	*E. coli*	GPT21-04	13	15	28
	*C. coli*	GPT21-05	15	15	30
	*C. coli*	GPT21-06	15	14	29
GPT 2022	*S. aureus*	GPT22-01	18	17	35
	*S. aureus*	GPT22-02	18	18	36
	*E. coli*	GPT22-03	18	19	37
	*E. coli*	GPT22-04	18	18	36
	*E. faecium*	GPT22-05	16	15	31
	*E. faecalis*	GPT22-06	15	15	30

^*a*^ Number of genomes considered for analysis for each strain for pDNA and BACT samples. The number of genomes is after the initial exclusion step, which was based on less than 95% match to the expected cgMLST or assembly deviating from the reference genome size by more than 10%.

**Fig 3:**
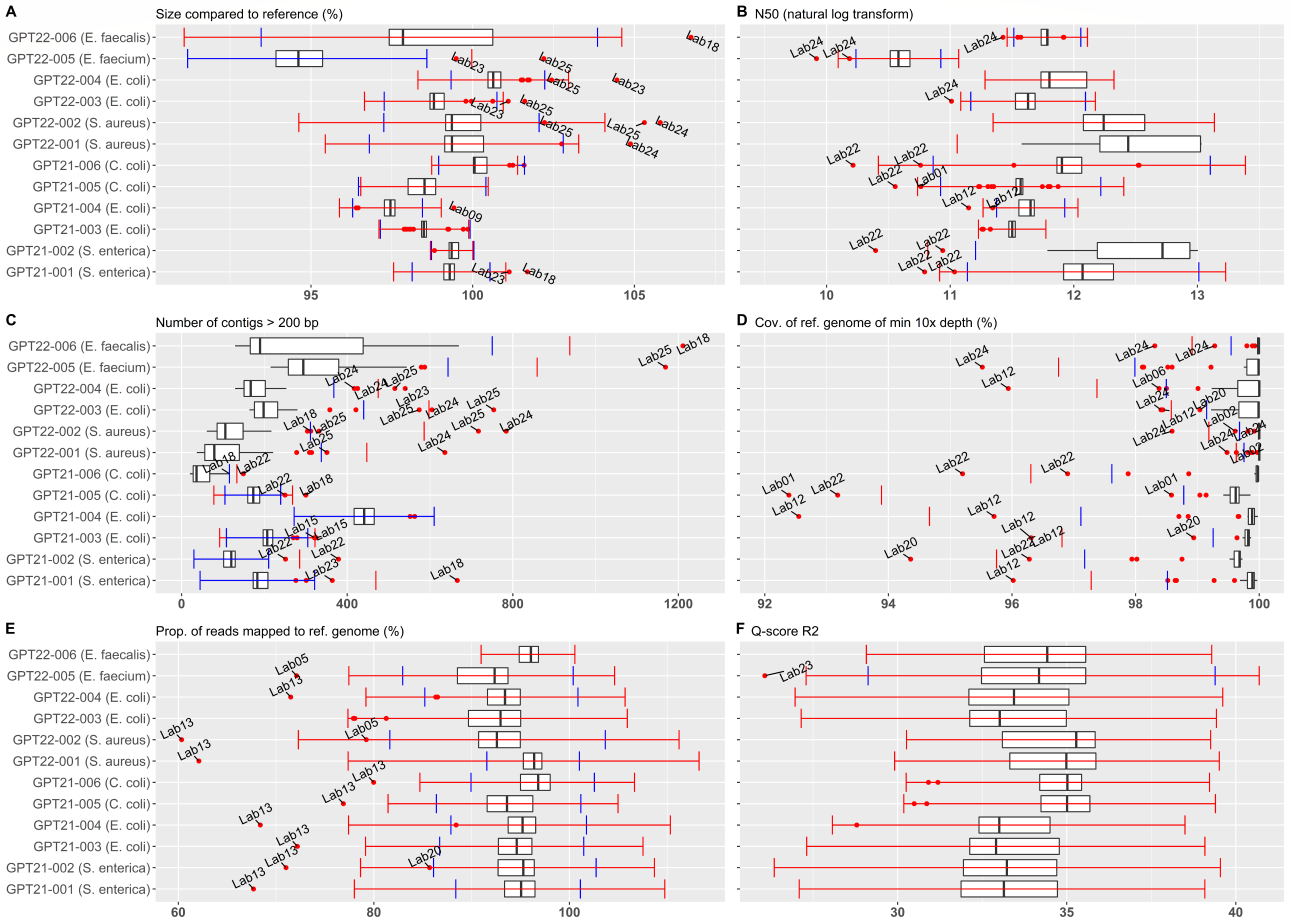
Boxplots of QC parameters. Quality parameters evaluated for all participants. For each parameter, the calculated outlier thresholds (red whiskers) and AQTs (blue whiskers) are shown along with boxplots for each isolate. Data points outside outlier and AQTs are labeled with submitting participant.

Outliers and genomes outside AQT were identified for each participating laboratory, presented in Fig. 3. Statistical values for each QC metric per species are available in Table S3. In 2021, 34 genomes were identified as outliers or outside AQT. These were mainly attributed to Lab12 (*n* = 6), Lab13 (*n* = 6), and Lab22 (*n* = 7). The remaining 15 genomes were from Lab01, Lab09, Lab14, Lab15, Lab18, Lab20, and Lab23 (each: *n* ≤ 4), mostly from BACT samples (22/34, 64.7%). The 2022 iteration had a total of 35 genomes identified as outliers or outside AQT. These were mainly attributed to Lab23 (*n* = 4), Lab24 (*n* = 11), and Lab25 (*n* = 8), while the remaining 12 genomes belonged to Lab02, Lab05, Lab06, Lab12, Lab13, Lab18, and Lab20 (each: *n* ≤ 3). In this iteration, the pDNA was the main cause (*n* = 18, 51.4%). The AQTs, including the log-transformed N50, an overview of isolates from each participant (Tables S4 and S5) and summarized kraken2 results are presented in the Table S6.

### Linear regression

Prior to pooling BACT and pDNA genomes, differences in distributions were tested. Among the tested parameters, only a “proportion of mapped reads” for isolate GPT22-03 and a “coverage of minimal depth 10× of reference genome” for GPT22-04 showed significant differences between distributions, with BACT genomes having a lower mean than those of pDNA (see Table S1).

The linear regression is visualized as heatmaps in Fig. 2. The “logN50” has a strong positive correlation with “N50” and “NG50” (see Fig. 2A and C). Positive correlation with assembly size metrics for 2021 isolates were observed, but primarily when excluding contigs less than 200 bp. Genomic coverage metrics of 5× and 10× showed strong positive correlation in the 2021 data and less but apparent correlation in the 2022 iteration. “Total number of contigs” correlated negatively, but the same tendency was not apparent for contigs less than 200 bp, though the 2021 data showed a weak correlation.

The metric “contigs > 200 bp” correlates strongly with size of assembly, though notably and in contrast to “N50,” excluding contigs of less than 200 bp reduces the strength of the correlation (see Fig. 2B). In the 2022 data set, the correlation is much more pronounced than the 2021 data set.

“Coverage of reference genome/chromosome” could not be calculated for GPT22-01 and GPT22-02 as all participants achieved 100% coverage, disregarding samples removed in the initial exclusion.

### Performance

Based on genomes removed by the initial exclusion criteria (see Table S2) or found to be outside AQT (see Fig. 3A through F), three laboratories were found to underperform, Lab22 in 2021 and Lab13 and Lab24 in 2022 (see Table 4). Lab22 had seven out of eight submitted 2021 genomes identified outside AQTs for “N50,” four of which also exceed AQTs for “Number of contigs > 200 bp,” both considered critical and uncorrelated QC metrics (Fig. 3B and C).

**TABLE 4:** Overview of laboratory performance^*a*^

	Percentage coverage of minimum 10× depth of the reference genome	N50	Percentage in size compared to the reference genomes	No. of contigs >200 bp	Proportion of reads mapped to the reference genome	Phred quality score (*Q* score forward and reverse)	Samples removed due to genome size deviating >10% from reference	Assigned MLST	<95% core MLST genes match	SNP difference >10 SNPs
Correlation group	1	1	2	2						
Year 2021										
Lab01	O	B							X	
Lab03										
Lab04										
Lab07							X			X
Lab09		B	B							X
Lab11										
Lab12	O	O			B					
Lab13		B			O					
Lab14	B									X
Lab15			O	O						
Lab16										
Lab17										
Lab18			O	O						
Lab20	O	B			B					X
Lab22	O	O	B	O						
Lab23			B	B						X
Year 2022										
Lab01										
Lab02	O									
Lab04										
Lab05	O				O					
Lab06	B									
Lab07										
Lab09										
Lab12	B							X	X	
Lab13					O		X			
Lab15										
Lab16								X	X	
Lab17										
Lab18			O	O			X			
Lab19							X			
Lab20	B									
Lab21										
Lab23			O	O		O				
Lab24	O	O	O	O						
Lab25			O	O			X			

^*a*^ Overview of participating laboratories each year and genomes failing a QC threshold. Parameters in the same correlation group have been indicated with a number and parameters not found to correlate left unnumbered. Gray shading has been applied to correlation group 2 columns to enhance readability. For each participant, it is indicated if at least one genome was found to be an outlier (O), was outside the AQT but not an outlier (B), or failed the minimal threshold (X). Participants identified as underperforming have their laboratory underlined.

Lab13 underperformed in the 2022 iteration due to two of their 12 genomes exceeding 10% variation in genome size. A further three of their submission were outliers in “Proportion of reads mapping to reference,” which were also the case for all of their 2021 submission (Fig. 3E). Lab24 had 11 out of 12 genomes outside AQT, four genomes below AQT for “N50,” five above AQT for “Number of contigs >200 bp,” six below “coverage of reference of min depth 10×” and two above the AQT for “Size of assembly compared to reference” (Fig. 3B through E). Other NRLs had genomes deviating outside AQTs, but not across critical or closely correlating QC metrics.

### Species-specific QC thresholds

Average minimal QC thresholds for each species were calculated for reference independent QC metrics for guidance in routine laboratory QC. For “N50,” based on the log transformed data, these were found to be 78,461 for *S. enterica*, 80,402 for *E. coli*, 59,044 for *C. coli*, 86,220 for *S. aureus*, 101,114 for *E. faecalis*, and 28,276 for *E. faecium*. Average thresholds based on “N50” without log transformation is available in the Tables S7 and S8. For “Number of contigs > 200 bp”, these were found to be 263 for *S. enterica*, 432 for *E. coli*, 168 for *C. coli*, 305 for *S aureus*, 716 for *E. faecalis* and 641 for *E. faecium*.

### SNP distance

For all submitted participant genomes, GPT21-006 (*C. coli*), GPT22-001 (*S. aureus*), GPT22-002 (*S. aureus*), GPT22-003 (*E. coli*), GPT22-005 (*E. faecium*), and GPT22-006 (*E. faecalis*) were all within 10 SNPs of corresponding reference. The GPT21-001-4 submissions had four, two, five, and 10 submitted genomes with more than 10 SNPs to the reference, respectively. For GPT21-005 (*C. coli*), we only observed three submissions below 10 SNPs, the remaining 27 having more than expected. For GPT22-004 (*E. coli*), we observed six submissions below the 10 SNP threshold, the remaining 30 having more SNPs than expected, ranging up to 56 SNPs to the reference genome. The majority of SNPs were found to originate from plasmid 2, accounting for 96.5% of SNP calls. SNP differences were calculated omitting plasmid 2, which reduced the number of differences between participant genomes and GPT22-004 (*E. coli*) to ranging from 0 to 2 SNPs. Full SNP matrices can be found in Tables S9 to S20; GPT22-004 (*E. coli*) SNPs with and without plasmid is found in Table S18.

## DISCUSSION

Overall, when we assessed the submitted genomes, we found them to be of high quality. In each iteration, a single participant (Lab22 and Lab24) was found to be underperforming, as they submitted samples, which were outside AQT for uncorrelated QC parameters. Both participants’ submissions lacked sufficient genomic coverage, indicating error in the DNA extraction or library preparation workflows. One additional participant in 2022 (Lab13) was identified as underperforming, due to two submitted genomes being removed in the initial exclusion step. This participant had all submitted genomes identified as outliers in 2021 (see Table S3), solely due to a low proportion of mapping reads. In 2022, this laboratory’s performance had worsened. Two isolates deviated by more than 10% in assembly size, and in two of the remaining genomes, nearly 40% of reads cannot be mapped to the reference, which suggests contamination. Kraken2 results indicated the contaminant to be an *Escherichia* spp. As the deviating genomes were from live cultures, the contamination likely occurred in workflows prior to library preparation.

In GPT 2021, we overall observed a higher number of SNPs than expected among participants. This is likely a consequence of the change in methodology used for constructing the reference genomes. The reference genomes for 2021 were closed with a hybrid assembly of ONT and Illumina reads, in contrast to the use of PacBio in other years. This assumption is supported by the lower number of SNP differences observed between genomes submitted by participants, which suggests a systematic error. Had the SNPs been from natural mutation, it is unlikely they would be shared across participants. Therefore, we suspect the cause to be assembly error due to low coverage regions or mapping errors. While the reference genomes are still expected to provide a much better representation of the actual genomic content compared to what can be achieved with either short- or long-read sequencing in isolation, the larger number of SNP calls suggests the expected threshold may have to be adjusted if hybrid assemblies are used in the future. Both mapping and assembly error may be reduced as ONT technology matures. We anticipate hybrid assemblies will improve as ONT technology moves closer to the quality of NGS technologies, in particular, with the newer chemistry and flow cell versions (31, 32).

In the 2022 genomes, GPT22-004 (*E. coli*) genomes were observed to have high SNP counts across nearly all participants (29/36, 80.6%). Further investigation revealed nearly all SNPs were called from plasmid_2 of the reference (see Table S18). The areas containing the SNPs had substantially higher coverage than flanking areas, indicating repeated sequences. Subsequent manual inspection confirmed the regions contained mobile elements. The high SNP counts are suspected to arise from high variability in these loci causing issues in the read mapping. We therefore chose to exclude SNPs from plasmid_2 in the evaluation, leading to no more than 0–3 SNP differences between reference and participant genomes.

The threshold calculations for “N50” and “number of contigs >200 bp” were based on the method describe by Kristensen et al. (21), though, here, we have used the median instead of the mean for estimating the mode of the distribution. This change was applied as these metrics have one-tailed distributions, meaning outliers can have a large effect on the mean.

In the GPT 2020, the “N50” metric provided very little discriminatory information because the variance affects the AQT and outlier calculation. “N50” can have increments of thousands of base pairs between genomes resulting in high variance. In this evaluation, “N50” data were transformed with the natural logarithm prior to setting thresholds, giving the “logN50” metric.

In the 2020 iteration, we suggested thresholds for “N50” of 20,000 and 25,000 for *E. coli* and *Campylobacter* spp., respectively (21). The minimal thresholds based on our updated methodology place “N50” at approximately 80,000 and 60,000 for *E. coli* and *Campylobacter* spp. respectively. Furthermore, we were able to set a threshold for *S. enterica* of approximately 75,000, which was not possible in our previous work. The higher “N50” thresholds may partly be explained by the change in initial exclusion criteria. The use of cgMSLT alleles is likely more discriminatory toward low-coverage genome submissions, which may produce more fragmented assemblies. Another likely factor is a higher genome complexity in the GPT 2020 isolates, as *E. coli* can have considerable intraspecies genomic variation (33). A general improvement among the participants may likewise explain the increase.

For species with large variances in genome size, such as *E. coli*, the suggested threshold of 80,000 may be excessive for complex or small genome variants within the species. For such species, it may be prudent to set intraspecies thresholds based on a subset of the population. However, such an approach needs to identify relevant genomic markers to efficiently classify genome complexity irrespective of whether a reference is available before it can be meaningfully implemented in routine laboratory usage.

For *Campylobacter* spp., the new suggested threshold is further improved by an increased number of submissions, as only one strain was used for the GPT 2020 suggestion. The results presented here are based on *C. coli*, meaning thresholds may not be sufficient for *C. jejuni*, though the two species are closely related (34). In *Enterococcus* ,however, there are large differences between *E. faecium* and *E. faecalis* as the thresholds for these species are more than 70,000 apart. *S. aureus* likewise has considerable differences between isolates.

Comparing thresholds for number of “contigs >200 bp” with those obtained in GPT 2020, we see general increases, with 37, 67, and 67 contigs for *S. enterica*, *Campylobacter* spp.. and *E. coli*, respectively. Overall, the thresholds are expected to be improved as the method makes less assumptions about the underlying distribution and is based on a larger number of samples and strains. The estimate for *E. coli* is much higher, largely due to isolate GPT21-004, which had generally high contig counts. Indeed, the median for this strain was 439 contigs, meaning the average threshold is likely ill suited for this specific strain. Identifying relevant criteria to further classify species with large intraspecies variation may be highly informative for setting useful thresholds for routine QC, thereby avoiding unnecessary re-sequencing.

Compared to previous suggestions for WGS QC thresholds, the European Committee on Antimicrobial Susceptibility Testing (EUCAST) subcommittee on WGS in AST suggested a maximal 1,000 contigs and “N50” above 15,000 to be of good quality, although an “N50” above 30,000 is preferred and less than 100 contigs considered achievable for genomes in the range 5–6 Mbp (20). The European Food Safety Authority (EFSA) mirrors the “N50” of 30,000 threshold in their WGS reporting pipeline (35). They further recommend a maximal 500 contigs for bacteria, but suggest 300 and 500 contigs as upper limits for *S. enterica* and *E. coli*, respectively (35, 36). The FDA’s GenomeTrakr network does not suggest N50 thresholds, but has suggested “number of contigs” thresholds for a number of pathogens, including 300 for *C. jejuni* and similar thresholds for *S. enterica* and *E. coli* as those suggested by EFSA (22).

For the isolates included in the 2020, 2021, and 2022 iterations of the GTP, “N50” thresholds appear overly inclusive as the average achieved is considerably higher for all strains. Even isolates showing considerable indication of contamination in their assembly size could achieve an “N50” of 30,000. Despite being a critical metric for WGS evaluation and generally mandatory when reporting assemblies in repositories, useful thresholds are not readily available for this metric.

Considering instead the “number of contigs,” EUCAST recommendations for thresholds of 100 or 1,000 contigs appear ill suited for *E. coli*. Even excluding contigs >200 bp, the median in this study was 150–450 contigs. This means that more than 50% of submitted *E. coli* genomes cannot meet the lower criteria, despite an expected genome size of 5 Mbp. Conversely, the higher threshold is overly inclusive, especially for the low count strains.

EFSA and GenomeTrakr suggestions for *E. coli* and *S. enterica* are very comparable to those found in this study, while the threshold for *C. jejuni* is almost twice the amount we suggest for *Campylobacter* spp. Our findings are based on *C. coli*, which may account for this discrepancy, or it could be that the selected isolates in the GPTs simply do not produce high numbers of contigs. However, it is possibly a result of insufficient coverage as Segerman et al. (37) illustrated that a low-GC content spp. needed higher depth to achieve full genomic coverage when using the now outdated Illumina Nextera XT chemistry. The suggested threshold for *E. coli* is likewise high compared to most of the isolates used in the GPTs, which may be improved by adjusting for genomic complexity as discussed above.

The linear regression showed that “contigs >200 bp” correlated well with “size of assembly.” Interestingly, the metric “average insert size” was not found to correlate in contrast to observations in the GPT 2020.

Excluding contigs less than 200 bp appears to weaken the correlation between “assembly size” metrics and “number of contigs >200 bp” and should therefore be avoided when evaluating sequencing runs.

“N50” and “logN50” correlate closely, meaning the change in AQT calculations should have little impact on established correlation groups. In stark contrast to GPT 2020 findings, “N50” metrics do not correlate strongly with “proportion of mapped reads.” While weak correlation might be interpreted from GPT 2021 data, no such tendency is evident in GPT 2022. These changes in correlation may be due to the changes in species, where GPT 2021 is more comparable to GPT 2020. Likewise, it may be a result of improvement among participants. No major differences were observed in the reported laboratory methodology, kits, and platforms between years; thus, their impact is thought to be less important. Nonetheless, the metric “proportion of mapped reads” will not be considered part of the “N50” correlation group in the 2021 and 2022 GPT iterations.

We implemented genomic coverage of differing minimal depth, as 1× depth provided little indication of overall quality. Most participants achieved close to 100% coverage of 1×, but areas with low coverage are prone to basecalling and assembly errors. While genomic coverage is simply a measure of read saturation across the genome, higher depth coverage describes the proportion of the genome which can reliably be used for analysis. Here we found that among the utilized coverage of minimal depths, 10 x provided the best correlation with “N50.” While 5× shows a comparable positive correlation, it evidently shows stronger negative correlation with “contigs >200 b,” which would put it into multiple correlation groups. Therefore, we chose to continue using the parameter “coverage 10× of the genome” for QC evaluation, as it showed slightly better correlation than “coverage 10× of the chromosome” to “N50” across isolates. Including plasmids is not expected to complicate the reliability of this measurement, as plasmids constitute relatively little of the total genomic DNA.

Inter-laboratory testing is of high importance for maintaining the quality of laboratory workflows. Laboratories with an accredited method for sequence data generation following the ISO/IEC 17025:2017 or ISO 23418:2022 (38, 39) are required to participate regularly in proficiency tests or inter-laboratory comparisons as part of their QC validation. While the GPT also has an AMR component, successful detection of AMR determinants does not necessitate high-quality sequence data. For laboratories conducting AMR detection, identification of such determinants may appear more relevant. However, AMR determinants present a small fraction of genomic information, and the ability to identify them will depend upon their genomic location, synteny, and copy number. Genomic analysis necessitates interpretation, making AMR determinants impractical for the evaluation of sequence quality. Here, we have identified laboratory underperformance from uncorrelated WGS QC parameters based on participant data (21). Comparison of QC parameters among laboratories validates a laboratory’s ability to produce sequence data of sufficient and comparative quality to peers.

A key limitation to consider for our findings is the limited number of samples, especially across different species. The same strain is usually not repeatedly sequenced outside WGS proficiency tests, meaning few collections of WGS data suitable for this type of analysis exist. Repeated sequencing is required to produce a reliable distribution of WGS QC results and effectively identify outliers. To establish reliable QC thresholds, it is necessary to increase the number of isolates employed in GPT initiatives. Increasing the number of participating laboratories, involved as well, can help negate the impact of outliers. Large variance among participants will affect thresholds, making them overly inclusive. In contrast, outliers due to exceptional performance is unlikely as most of the applied quality metrics have an upper or lower limit.

The lack of unanimously accepted species-specific QC thresholds to assess whether the acquired sequences are fit for use in downstream *in silico* analyses is a limitation toward the routine implementation of WGS in AMR surveillance. GPTs and similar initiatives can play a pivotal role in this endeavor, as repeated sequencing of a strain across workflows is necessary to understand what constitutes good quality. This is particularly relevant as the declining price of WGS makes it more accessible for genomic surveillance.

### Conclusions

Participants in the EURL-AR network in general performed well, producing overall high-quality data. Contamination is still a significant challenge in routine sequencing, which underpins the necessity of inter-laboratory testing and continual evaluation of wet-lab practices.

Building on a revised GPT 2020 methodology, which was applied to GPT 2021 and GPT 2022 genome submissions, we suggest revised species-specific QC thresholds for routine sequencing: *E. coli*, a minimum “N50” of 80,000 (increased from 20,000) and a maximum “number of contigs >200 bp” of 450 (increased from 265).

*Campylobacter* spp., a minimum “N50” of 60,000 (increased from 25,000) and a maximum of 175 “contigs > 200 bp” (increased from 100).

*S. enterica*, a minimum “N50” of 75,000 and a maximum 275 “contigs >200 bp” (increased from 225).

We suggest initial thresholds for “N50” of 85,000, 100,000, and 30,000 and thresholds for “contigs >200 bp” of 300, 725, and 650 for *S. aureus*, *E. faecalis*, and *E. faecium*, respectively.

Compared to EUCAST, EFSA, and GenomeTrakr recommendations, the “N50” QC thresholds suggested here appear better suited to the current proficiency of laboratories and available technology. The suggestions for “number of contigs” are comparable and possibly improved compared to previous guidelines due to changes in library preparation chemistry.

## Data Availability

Sequence data from the GPT 2021 and 2022 have been submitted to the European Nucleotide Archive (ENA) under project accession PRJEB65174 and PRJEB65175, respectively. Closed reference genomes are available under the accessions listed in Table 1.

## SUPPLEMENTAL MATERIAL

Figure S1 msystems.00160-24-s0001.docx Map of participating countries.

Supplemental tables msystems.00160-24-s0002.xlsx Tables S1-S20.

ASM does not own the copyrights to Supplemental Material that may be linked to, or accessed through, an article. The authors have granted ASM a non-exclusive, world-wide license to publish the Supplemental Material files. Please contact the corresponding author directly for reuse.
