# Supplementary material for: Whole-genome sequencing for antimicrobial surveillance: species-specific quality thresholds and data evaluation from the network of the European Union Reference Laboratory for Antimicrobial Resistance genomic proficiency tests of 2021 and 2022
Source: mSystems. 2024 Aug 6;9(9):e00160-24. doi: 10.1128/msystems.00160-24 (PMC11406893; doi:10.1128/msystems.00160-24)
Supplement: Figure S1 — Map of participating countries. [file msystems.00160-24-s0001.docx]

**Figure S1: Participation**


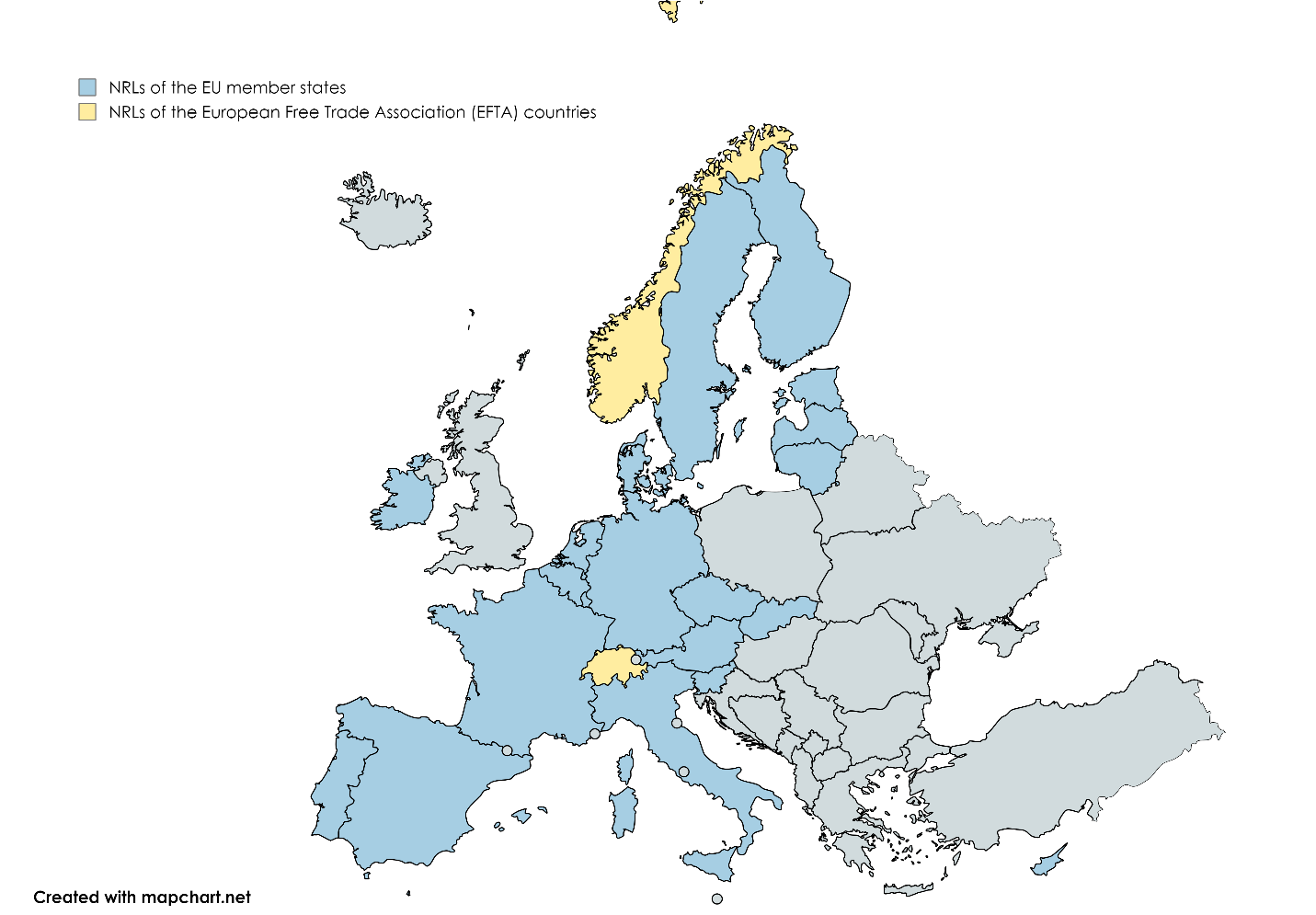


Figure S1: Countries participating in 2021 and 2022 iterations of the EURL-AR Genomic Proficiency Test.
